# Parental Engagement in Identifying Information Needs After Newborn Screening for Families of Infants with Suspected Athymia

**DOI:** 10.1007/s10875-024-01678-w

**Published:** 2024-03-08

**Authors:** Evey Howley, Maarja Soomann, Alexandra Y. Kreins

**Affiliations:** 1 Department of Immunology and Gene Therapy, Great Ormond Street Hospital for Children NHS Foundation Trust, London, UK; 2 Division of Immunology and the Children’s Research Centre, University Children’s Hospital Zurich, University of Zurich, Zurich, Switzerland; 3 Infection Immunity and Inflammation Research and Teaching Department, University College London Great Ormond Street Institute of Child Health, London, UK

**Keywords:** Newborn screening, severe combined immunodeficiency, congenital athymia, thymus transplantation, patient and public involvement and engagement

## Abstract

Congenital athymia is a rare T-lymphocytopaenic condition, which requires early corrective treatment with thymus transplantation (TT). Athymic patients are increasingly identified through newborn screening (NBS) for severe combined immunodeficiency (SCID). Lack of relatable information resources contributes to challenging patient and family journeys during the diagnostic period following abnormal NBS results. Patient and Public Involvement and Engagement (PPIE) activities, including parental involvement in paediatrics, are valuable initiatives to improve clinical communication and parental information strategies. Parents of infants with suspected athymia were therefore invited to discuss the information they received during the diagnostic period following NBS with the aim to identify parental information needs and targeted strategies to address these adequately. Parents reported that athymia was not considered with them as a possible differential diagnosis until weeks after initial NBS results. Whilst appropriate clinical information about athymia and TT was available upon referral to specialist immunology services, improved access to easy-to-understand information from reliable sources, including from clinical nurse specialists and peer support systems, remained desirable. A roadmap concept, with written or digital information, addressing parental needs in real time during a potentially complex diagnostic journey, was proposed and is transferrable to other inborn errors of immunity (IEI) and rare diseases. This PPIE activity provides insight into the information needs of parents of infants with suspected athymia who are identified through SCID NBS, and highlights the role for PPIE in promoting patient- and family-centred strategies to improve IEI care.

**Supplementary Information:**

The online version contains supplementary material available at 10.1007/s10875-024-01678-w.

## Introduction

**Fig. 1:**
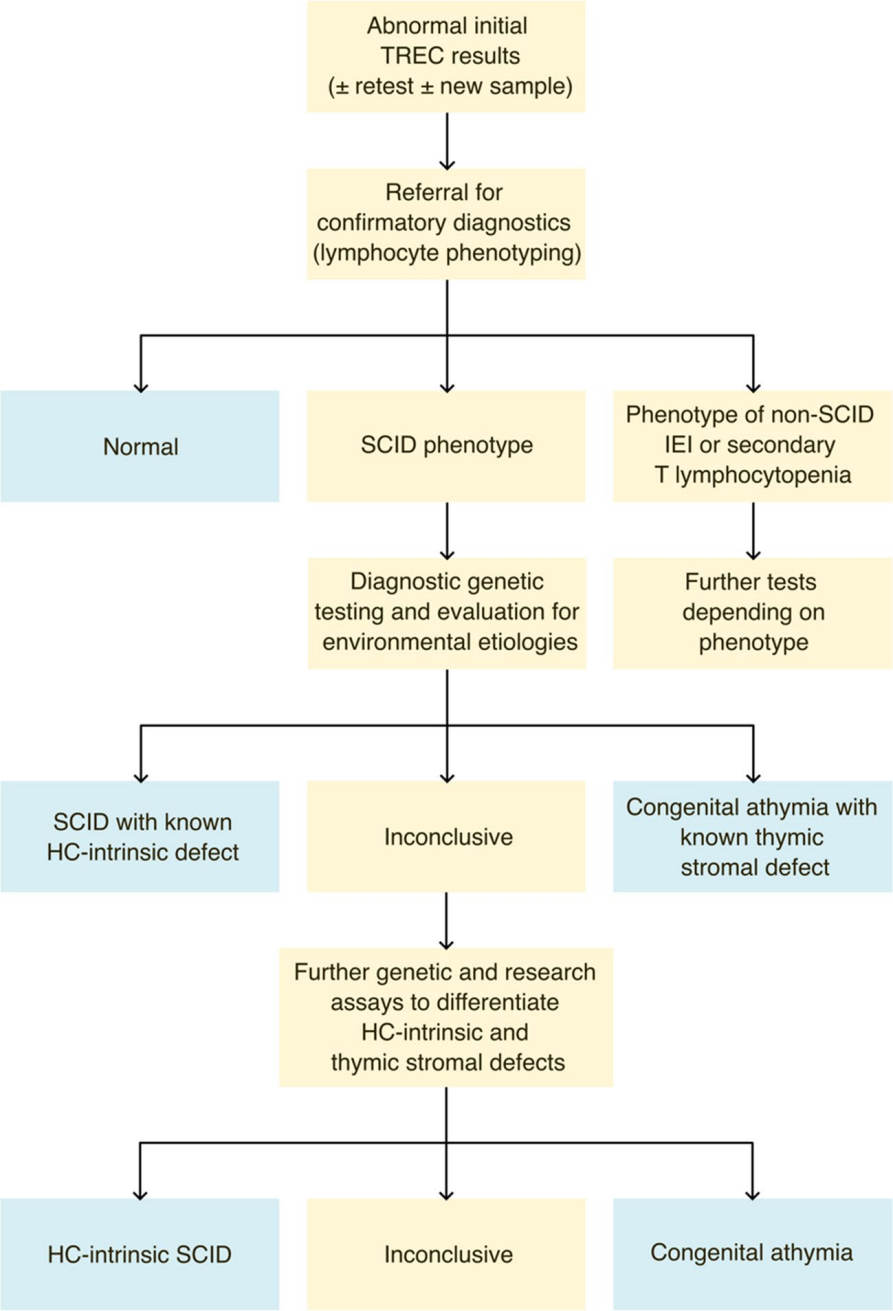
Simplified algorithm for the diagnostic process prompted by abnormal findings in TREC-based NBS for SCID and T lymphocytopaenia. IEI, inborn errors of immunity; HC-intrinsic, haematopoietic cell-intrinsic; SCID, severe combined immunodeficiency; TREC, T lymphocyte receptor excision circles

Severe combined immunodeficiency (SCID) is fatal in the first year of life unless diagnosed early and corrected, usually by allogeneic haematopoietic stem cell transplantation (HSCT) [1]. SCID newborn screening (NBS) enables diagnosis shortly after birth in asymptomatic infants by quantifying T lymphocyte receptor excision circles (TREC) in routinely collected dried blood spots (DBS) [2–4]. Universal SCID NBS was first established in the United States (US) [5] and is being implemented in many other countries [6], with superior outcomes after HSCT in patients identified through NBS compared to infants diagnosed based on clinical presentation [7]. Following SCID NBS algorithms, infants with repeatedly abnormal TREC values are referred for confirmatory diagnostic testing by specialist immunology services (Fig. 1) [6]. SCID is suspected in infants with abnormal lymphocyte subsets, specifically CD3^+^ T lymphocyte counts < 300/µL and/or less than 20% of CD3^+^CD4^+^ lymphocytes with naive cell surface markers [8]. Depending on B and NK lymphocyte counts a diagnosis of T^−^B^+/low^NK^+/low^ SCID is made, and antimicrobial prophylaxis and isolation are initiated [9, 10]. Genetic investigations to confirm the underlying condition before proceeding with corrective treatment are typically based on the analysis of gene panels including all known SCID genes and other relevant candidate genes, and are increasingly done by next-generation-sequencing (NGS) approaches [8, 9]. Despite expanding NGS access, approximately 10% of patients with suspected SCID remain without a genetic diagnosis [11].

Whilst very sensitive [12–14], TREC-based screening is not specific to SCID; it also identifies non-SCID T lymphocytopenia, including inborn thymic stromal cell defects associated with selective failure of T lymphocyte development [8, 14, 15]. Impaired thymus organogenesis is most commonly due to 22q11.2 deletion syndrome (22q11.2DS) and patients variably display syndromic features, including thymic hypoplasia, hypoparathyroidism and conotruncal congenital heart defects, a clinical triad referred to as DiGeorge Syndrome (DGS) [16]. A minority of 22q11.2DS patients suffer from thymic aplasia resulting in congenital athymia with a T^−^B^+^NK^+^ SCID phenotype and absent thymic output, characterised by negligible TREC levels and less than 5% of T lymphocytes with a naïve phenotype [17, 18]. Athymia is also seen in other rare, syndromic disorders (Supplementary Table S1) [18–52]. It is a life-limiting condition and athymic patients are susceptible to severe infections and immune dysregulation. They require corrective treatment with thymus transplantation (TT), a highly specialised procedure [17, 18]. Due to the variable clinical penetrance of the syndromic features, a thymic defect may not initially be suspected, but thanks to NBS, athymic patients are increasingly identified early after birth [14, 53, 54]. Genetic testing in patients with suspected athymia include cytogenetic studies to detect chromosomal abnormalities and/or gene panel testing [8, 9]. If no genetic aetiology is identified, first trimester *in utero* exposure to environmental toxins should be considered (Supplementary Table S1). Early diagnosis and early TT have recently been shown to improve outcomes, including better recovery of T lymphocyte immunity earlier after transplantation [53].

In Europe, TT is offered at Great Ormond Street Hospital (GOSH) in the United Kingdom (UK) [17, 47, 53]. When suspecting athymia, the GOSH TT program can provide specialist advice for clinicians and families [55], but a lack of awareness of access to TT programs has been reported [56]. The low incidence of congenital athymia (0.48 per 100 000 births in a recent NBS study in Germany [14]) and the geographical spread of the patients likely contribute to this. There are also no registered national or international patient advocacy groups specifically for athymic patients and their families. SCID is more common than congenital athymia (1.5 to 4.0 in 100 000 births according to recent studies) [12, 14, 57], yet little is known on the information needs for families of infants diagnosed with SCID through NBS [58, 59], and no data is available on the information needs for the subgroup of families of infants with suspected athymia. For this reason, a patient involvement and engagement project to aid the understanding of information needs in this context and to guide the development of targeted resources to improve patient and family education was initiated at GOSH, involving families who have lived experience. The following narrative highlights information needs as presented by families of infants with confirmed or suspected athymia, specifically during the diagnostic period following an abnormal NBS result.

## Methods

### Patients and Families

Families with newborns identified through NBS and referred to the TT program at GOSH between 10/2019 and 10/2023 with suspected athymia (Table 1), either for further investigations and/or treatment with TT, were considered for participation. Parents of patients who were alive and stable after treatment were invited via email by the GOSH TT clinical nurse specialist (CNS) or their referring clinician to get involved in focus groups or to provide written feedback in response to questions aimed at understanding information needs during the diagnostic period of suspected athymia after NBS (Supplementary Table S2).

**Table 1:** Diagnosis and therapeutic outcome for patients referred to Great Ormond Street Hospital between 10/2019 and 10/2023 with suspected congenital athymia following an abnormal NBS result

Diagnosis	Therapeutic outcome			
	TT	HSCT	Supportive care	Palliative care
DGS phenotype*	13		5	6
T^−^B^+^ NK^+^ SCID	4	2		
OFCS2	1			
hetFOXN1			2	
All	18	2	7	6

*DGS *DiGeorge Syndrome (* genetically confirmed); *hetFOXN1 *heterozygous mutation in Forkhead box protein N1, *HSCT *haematopoietic stem cell transplantation, *OFCS2* otofaciocervical syndrome type 2, *SCID *severe combined immunodeficiency, *TT *thymus transplantation

### Parental Engagement

Group or individual sessions were facilitated through videoconference calls by the GOSH TT CNS, who was known to most families and has experience facilitating parent focus groups. Language interpretation support was provided voluntarily by members of staff at GOSH. Videocalls were planned to last up to 30 min. Short, open-ended questions were shared beforehand to guide parent discussion (Supplementary Table S2). Informed consent was obtained from all participating families.

### Identification of Information Needs

Information needs were identified by the authors after completion of the videocalls and from the written feedback. Anonymised parental quotes were selected to illustrate information gaps and highlight opportunities for improving information resources.

## Results

### Identification of 33 Patients Diagnosed through NBS

**Fig. 2:**
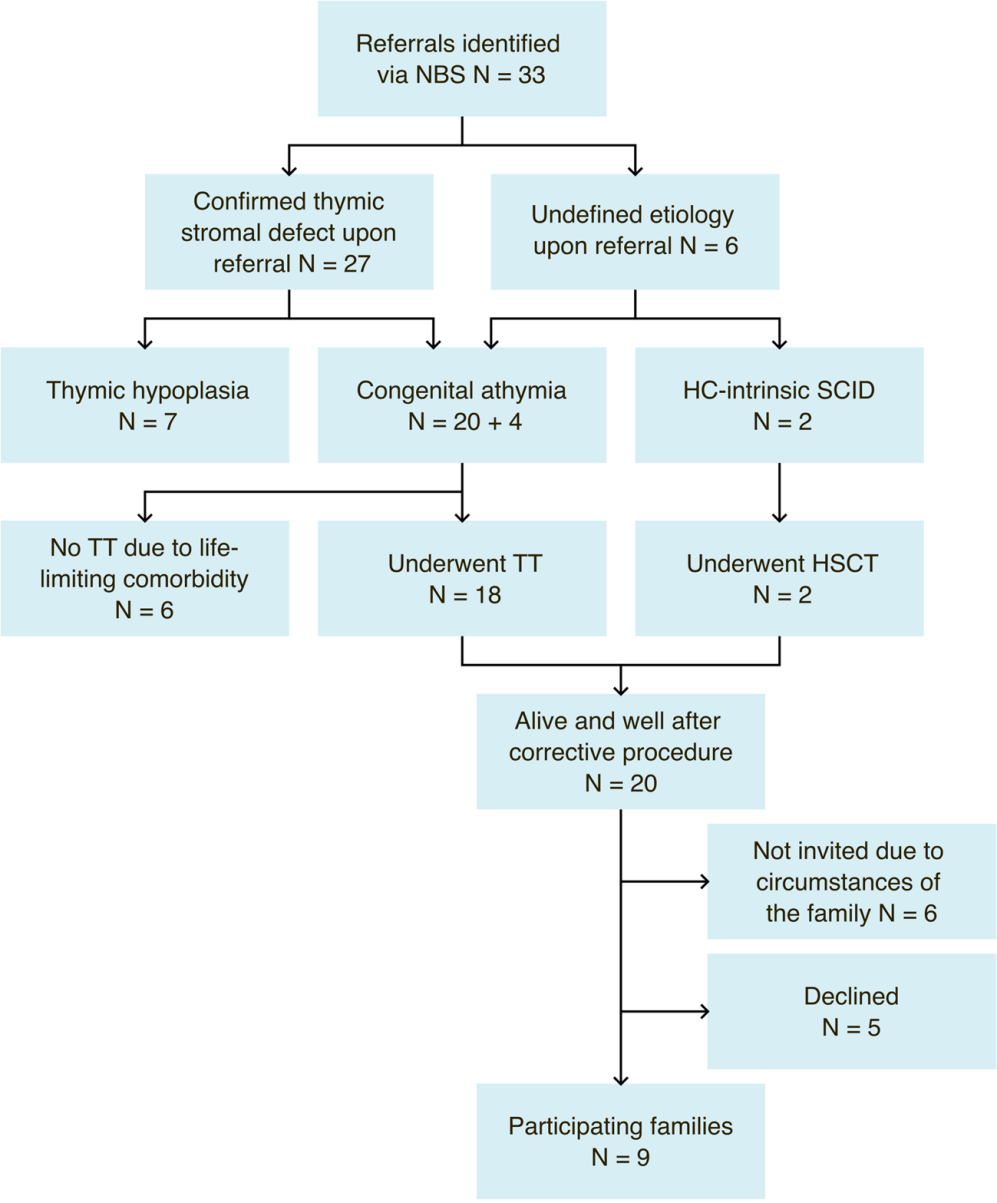
Participant flow diagram. HSCT, haematopoietic stem cell transplantation; N, number; NBS, newborn screening; SCID, severe combined immunodeficiency; TT, thymus transplantation

Over a four-year-period (10/2019-10/2023), 33 patients identified through NBS from 26 centres across 19 countries (23 patients from UK and European Union (EU) and 10 from non-EU countries) were referred to GOSH with suspected athymia. All patients were referred by paediatric specialists providing care for SCID patients, of whom 58% had previous experience referring patients to the TT program. 27/33 referrals (82%) had a thymic defect with a known molecular aetiology for which successful treatment with TT has been reported (Fig. 2). Immunological investigations confirmed athymia in 20/27 patients. 7/27 patients were shown to have residual thymic output and were diagnosed with thymic hypoplasia rather than athymia. 6/33 patients (18%) were referred with genetically undefined T^−^B^+^NK^+^ SCID requiring further investigations before deciding the most appropriate corrective procedure, HSCT or TT. These investigations included broader genetic testing with agnostic analyses [60, 61] and diagnostic research assays to test the ability of their CD34^+^ haematopoietic stem and progenitor cells to differentiate into CD3^+^ T lymphocytes in vitro to help distinguish haematopoietic cell-intrinsic defects from thymic defects [62–64]. Overall, 18/33 patients were accepted for TT and 2/33 underwent HSCT. Six athymic infants had life-limiting co-morbidities and received palliative care.

### Parental Involvement and Engagement

By 10/2023, all 20 patients were alive after their corrective procedures. Considering clinical and personal circumstances, 14 families were invited to participate in the proposed patient involvement and engagement activities. 9/14 (64%) families agreed to participate, 7/9 by videocall and 2/9 by providing written feedback. Demographic and clinical details of these nine patients are summarised in Table 2. Patients were under the care of nine different specialist centres in eight countries (6/9 in UK and EU, 3/9 in non-EU countries), including five centres that had previously collaborated with the TT program. 4/9 patients with a confirmed thymic defect upon referral underwent TT at a median age of 101 days (d) (range: 57-299d) with a median follow up time of 28 months (m) (range: 18-40 m). The remaining 5/9 patients were referred with a T^−^B^+^NK^+^ SCID phenotype but no known defect. 3/5 patients had syndromic features and one patient had previously been treated with HSCT. Of these 5 patients, 3 underwent TT (median age 325d, range: 195–669d; median follow up 9 m, range: 1.5-10 m). The remaining 2 underwent HSCT in their referring centres. A mixture of mothers, fathers or both parents (*N* = 11) got involved in the activities.

**Table 2:** Demographics of infants diagnosed via newborn screening with suspected congenital athymia whose families got involved in the Patient and Public Involvement and Engagement activities

Molecular diagnosis at referral (N)	Median age in days at referral to GOSH (range)	DGS features (N)	Additional genetics* (N)	In vitroT-cell differentiation assay (N)	Treatment (N)	Median age in days at transplantation (range)
DGS 22q11.2DS: 2 CHD7: 2	36 (5 to 78)	4	0	0	TT: 4	101 (57 to 299)
Undefined T^−^SCID: 5	55 (27 to 346)^#^	3	4	5	TT: 3 HSCT: 2	195 (105 to 673)

^*^genetic analyses beyond routinely available clinical testing; ^#^1/5 patients received previous HSCT; *CHD7 *Chromodomain-helicase-DNA-binding protein 7, *DGS *DiGeorge Syndrome, *DS *deletion syndrome, *HSCT *haematopoietic stem cell transplantation, *N *number, *SCID *severe combined immunodeficiency, *TT *thymus transplantation

### Identification of Information Needs

#### Initial NBS Results

7/9 families had received another clinical diagnosis before being informed of the abnormal NBS result. These infants were hospitalised due to prematurity (*N* = 3), cardiovascular (*N* = 2) or respiratory instability (*N* = 1) and feeding issues due to a cleft palate (*N* = 1). 2/9 newborns were at home, although one was admitted with hypocalcemic seizures shortly after receiving the NBS result and was diagnosed with primary hypoparathyroidism. Despite the diverse clinical situations, all families shared a similar initial experience. They all received the NBS result within the first days–weeks of life, leading to immediate changes in their child’s management to reduce infectious risks. All families acknowledged the importance of NBS in keeping their children safe, saying *“If (our child) hadn’t had that NBS, (they) probably would not be here now”*; *“(Our child) is still infection-free and I think it wouldn’t be possible if we were not isolated”.* Depending on the centres and departments, NBS results were shared by different specialists, primarily neonatologists and immunologists. Three families reported that clinicians initially suggested the results were probable errors, saying: *“The clinicians (neonatologists) said this is maybe because she is premature, it’s probably nothing”*; “*We have to check if it’s right or not…”*.

7/9 parents reported some degree of difficulty understanding the meaning of the NBS result, with limited information being provided and parents expressing confusion: “*They (neonatologist) told me like I should know what T cells are … I had no idea”*; *“It was all kind of gobbledegook”;* and “*After the phone call, I typed into Google «immune problems in newborn screening», and it came up with SCID, so I was reading about it and you know the first thing that you read is about bone marrow transplantation (BMT), and really I didn’t know what BMT was, but when you hear the word transplantation, you’re like oh my god and then it says, babies can’t survive”*. Two families reported that the explanation they were given referenced one of the earliest infants diagnosed with SCID: “*I was told he has flagged on the NBS for SCID, this is a severe immunodeficiency and you may know it as Bubble Boy disease”*; *“You know the Bubble child”*.

#### Differential Diagnoses

All families reported that their child initially received a diagnosis of SCID (including one patient with a prenatal diagnosis of 22q11.2DS), recalling: *“They (neonatologist) came in with a piece of paper printed out and it just had SCID information, that was it”*; *“Our doctor (neonatologist) told us our child had a SCID-level immunodeficiency”.* Only one parent reported that SCID and athymia were both mentioned as possible diagnoses from the start: *“They (immunologist) mentioned both possibilities, but they said that probably the SCID thing is more likely than the other diagnosis… It was made clear at this point that further tests were necessary in order to try to narrow down the diagnosis”*. HSCT was the primary treatment option reportedly discussed at the start of the diagnostic journey: *“We were told (our child) will need a BMT, we all were immediately sent for tissue typing”*.

The differential diagnosis of athymia and TT were not discussed with 8/9 families until weeks later or sometimes even longer if further investigations were necessary to confirm a thymic defect rather than a haematopoietic cell-intrinsic defect: *“On the day of the genetic diagnosis two doctors (neonatologists) came in… Your child has DiGeorge Syndrome… You have to go to London for a thymus transplant… We were actually totally surprised as nobody really had talked about it before*”; *“Thymus came weeks after”.* Two families reported they felt clinicians suspected their child may have athymia but initially did not share that with them: *“For me, it was still SCID and the doctors were probably already thinking about athymia but they didn’t tell us… We were really surprised… That was not the diagnosis we were prepared for”*; *“Congenital athymia came weeks after that (NBS result). It (the differential diagnosis) may have been available sooner, but it certainly wasn’t available for us as a family at that point in time. I would say that it was kept behind closed doors with Immunology while they (neonatologists) were looking at solutions”*. Both families reported that they would have liked to have had this information earlier, even if it was still just a possibility: *“Maybe it would have been better to kinda have been open about it, to say it also might be this… It would have been good to have been a little more prepared”*. Another family reported how they researched both options independently and had presented the idea of athymia and TT directly to their medical team: *“What about a thymus (transplant)? And they (immunologist) were like no, it won’t be a thymus (transplant), he will definitely need a BMT”*.

Parents shared mixed responses about the information they received once congenital athymia was introduced, expressing different levels of information needs. One parent was positive, saying: *“It was well explained by the doctor in charge (immunologist), she took the necessary time to get us through the explanations”* and “*For us, it was important to focus on one problem at a time… They (immunologist) made a (metaphorical) roadmap for us”*. Another parent found the experience more challenging: “*I think he (immunologist) was trying to explain it, but it wasn’t like I understood. I didn’t understand anything, it was all like big scientific words. I’m not very good at taking information in. I have to have someone really sit down and explain things to me”*. Families whose children were hospitalised due to clinical needs reported feeling overwhelmed at the time, and that they would have liked simple, basic information at the start: *“We were totally overwhelmed with everything”*; *“This is going to sound so basic: I got handed an A4 piece of paper about SCID, I would have loved having the same about congenital athymia”*.

#### Diagnostic Pathway in Genetically Undefined T Lymphocytopaenia

5/9 infants had genetically undefined T lymphocytopenia requiring investigations beyond routinely performed genetic and immunological tests (Table 2). 4/5 parents were positive about the access to and the use of diagnostic research assays through either the GOSH TT program or research laboratories, saying they received appropriate levels of information, with teams collaborating well for the benefit of their child: *“They worked together well”*; *“They always kept us updated”* and “*We understand what this investigation is”*. Parents also highlighted challenges relating to the extended time completing these tests required: “*There were months of uncertainty… Doctors were uncertain… So, without a gene, what are our choices… Will (our child) reach (their) first birthday?*”; *“We were waiting for ten weeks for information. It’s a really long time and you sit there in the hospital, and you don’t know anything and think, what are they waiting for? … We felt forgotten, lost in Googleland*”. One family expressed opportunities to improve their child’s care were missed, as they felt that access to these research assays had been delayed: *“It should have been considered more and it should have been discussed as a possibility with us… I guess it comes down to enough scientific backing”*. One parent, whose child was found to have a likely disease-causing defect which they inherited from their mother, specifically reported this had provided access to information to improve her own health and wellbeing: *“They (Thymus Transplantation team) found out (my child) had a genetic problem, but then they also found out that I have it too, and it’s like I have been fighting my whole life for answers”*.

#### Availability of Information on Athymia and TT

Parents reported challenges to accessing reliable, relatable information specifically on athymia and TT. 7/9 families said the internet was initially their main source of information: *“We got little to no information how the future would be like, would our (child) die, how soon, is it treatable. We were confused and had to google for information”*; *“Information about SCID and BMT is easy to understand, but the TT information, about this, it is not easy to find on the internet”*. For families who were treated by clinicians with previous experience of managing an athymic patient, the information shared was more positively reported: *“They did a great job, they explained us everything they could”*. In contrast, where clinicians had no previous experience, families expressed more uncertainty: *“We had thousands of questions, but the doctors (neonatologists) couldn’t answer them”; “It was like, almost, don’t ask too many questions”*. Upon referral to their primary immunologist or to the TT program, families reported the information improved and they felt reassured: *“They kind of explained everything a lot better really, they knew exactly what they were talking about, I felt a lot better after I spoke to them”.*

#### Access to Support Networks

Three families mentioned having contact with other families whose child had previously undergone TT. They reported this provided them real life, tangible information, on occasion in their native language, from peers who could empathise with them: *“We felt so alone, so unique, so different from all the other parents I knew… I remember writing with her as I was staying in (hospital) and (her child) had the transplant a year before us, so she knew a lot and it was great having a person speaking the language I speak”; “I found a contact on Instagram to (another parent) and she told me about Facebook groups and a TT support group and the SCID Angels for Life group and there is a lot of information that helped us so much”*. Families who did not access peer support at the time still feel this is an important information resource: *“That would have been a good thing if we had known that there might be other parents who already went through the process, because that’s a different perspective than the perspective of the medical teams”*. One parent suggested that they would have liked these resources to be presented more formally: *“Websites on the internet, contact with other parents, more Facebook groups but more official”*. Families also highlighted the importance of access to a CNS, including the TT program’s CNS, for information and support: *“I wrote a lot with (CNS) … At the time we had (our doctor) but we didn’t have this relationship that I felt always comfortable asking thousands of questions every time I had them”; “(CNS) who we have here, is incredible, absolutely amazing and they do all the background work … I don’t think you can do without an immunology clinical care nurse”*.

A comprehensive list of parental quotes relating to these 5 themes is provided in Supplementary Table S3.

## Discussion

Patient and Public Involvement and Engagement (PPIE) activity, including family involvement in paediatrics, is increasingly recognised in practice, policy and research as a fundamental requirement when setting priorities and designing services to improve patient care [65–68]. It provides opportunities for collaborative working, increasing patient choice and shared decision making [66, 67, 69], but even when clinical innovation is manifest and patient advocacy groups are active, there is disparity in the implementation of PPIE activities, most being delivered in research [67, 70, 71]. Across the field of NBS, PPIE work has exposed challenges for clinicians in patient and family counselling on rare diseases outside of specific specialist services, which can contribute to compromising patient and family journeys, with parents on occasion reporting the diagnostic period following NBS a lasting traumatic experience [72, 73]. In SCID NBS, abnormal results have been reported to cause stress, anxiety and fear to the parents of infants diagnosed with SCID and other T-lymphocytopaenic defects [59, 74, 75]. Congenital athymia is a rare T-lymphocytopaenic condition which is increasingly diagnosed in the context of SCID NBS [14, 53]. As PPIE initiatives involving parents in developing meaningful and easy-to-understand information resources have been shown to better address specific needs of the target audience [58, 72], we proposed a first engagement activity with parents whose children received a suspected diagnosis of congenital athymia after NBS.

Whilst families expressed gratitude that NBS contributed to early diagnosis and initiation of protective measures for their child, most reported confusion and anxiety after receiving the initial NBS results, with difficulty in understanding the implications. The information they received centred on SCID and HSCT, with the differential diagnosis of congenital athymia and its treatment with TT not being discussed until several weeks after. In retrospect, despite most having felt overwhelmed in the early stages, parents would have welcomed earlier, and more open discussion of various alternative diagnostic and therapeutic options being considered. Whilst information received from clinicians experienced in TT was reported to be satisfactory, communication from clinicians naïve to TT, including neonatologists but also primary care immunologists, tended to foster uncertainty and overall, information materials about athymia and TT were limited. Parents resorted to independent internet searches leading to poor quality and unrelatable information, which is not uncommon after abnormal NBS results [58, 72–74]. Parents expressed a need for basic, unambiguous information from trusted sources, preferably in written format. Access to CNS was reported as an important source of reliable information and was highly valued by families. Additionally, families who interacted with peers, reported this to be another important source of information, reassurance and comfort. Peer-to-peer support is often highlighted by parents in similar circumstances as a positive factor [72, 75, 76], and even those who did not directly access peer support, affirmed this as a useful and desired opportunity.

This PPIE activity highlighted several opportunities for improving information resources for parents during this diagnostic period. Firstly, efforts to raise clinicians’ awareness of the differential diagnosis of athymia and the availability of its appropriate treatment with TT are crucial. Whilst first-line clinicians delivering NBS results, including neonatologists, are more familiar with SCID than with congenital athymia, parents have expressed a clear desire for early knowledge on the differential diagnoses to aid preparedness. In this context, information on the relevance of research tests to differentiate between haematopoietic cell-intrinsic and thymic T-lymphocytopaenic defects in genetically undefined SCID should be emphasised among clinicians. Whilst these assays may not always be readily available, families whose infants have undergone these tests, consistently reported positive experiences, regardless of final diagnosis and treatment. To further improve overall patient and family experience and education, easy-to-understand information material about athymia and TT should be developed for parents, with involvement of families with lived experience in the design process. The GOSH TT program has previously involved families in the conception and design of a detailed frequently asked questions factsheet and a bespoke storybook resource for families in the preparation stage for TT [77]. For parents of infants under investigation for T-lymphocytopaenic IEI, who are at the beginning of the diagnostic and therapeutic journey, a simpler “roadmap” that delineates the necessary steps after confirmation of an abnormal NBS result would be a more appropriate tool. The roadmap concept has the advantage that it can highlight differential diagnoses and subsequent treatment pathways, whilst breaking down this potentially lengthy and complex journey and reducing the overwhelming burden associated with multiple diagnoses and uncertainty. Developing such resource in collaboration with families would ensure that it meets parents’ needs in terms of both content and style and that translation of complex information into simpler terms is prioritised [72]. The roadmap approach could be used in similar challenging diagnostic journeys in IEI and other rare diseases. While written documents were referenced by parents, these resources could equally be developed in a digital format.

Parents also emphasised the importance of access to trustworthy support systems. Development of the above documents in partnership with families and clinicians in referring centres, would reassure new parents that the information they receive has come from a trustworthy and relevant source. Among clinicians, parents recognised CNS as important and relatable sources of reliable information. CNS have been associated with long-term improved patient psychological wellbeing and satisfaction, information provision, and service co-ordination [78, 79]. Earlier referral to specialist Immunology services will facilitate access to Immunology CNS or the TT programme’s CNS sooner in the patient journey. Additionally, as peer support was seen uniformly as important, easy-to-find patient networks could make the journey easier for families. Currently no dedicated advocacy group exists for congenital athymia, however given this growing cohort, the development of an official support and advisory council could be considered in partnership with medical institutions and parent advocates. Such networks have been shown to be successful in similar instances [69, 76].

This first PPIE activity with parents of infants with a suspected diagnosis of congenital athymia after NBS provides unique insight into their information needs, promoting targeted strategies for improved communication, education and resource provision for families and for clinicians delivering NBS results. It also further highlights the role for PPIE in improving care in the fields of IEI and other rare diseases.

### Supplementary information

Below is the link to the electronic supplementary material.

ESM 1 (DOCX 86.5 KB)

## Data Availability

The data stored within the firewall of UK National Health Service are available on request from the corresponding author.
