# Supplementary material for: Parental Engagement in Identifying Information Needs After Newborn Screening for Families of Infants with Suspected Athymia
Source: J Clin Immunol. 2024 Mar 8;44(3):79. doi: 10.1007/s10875-024-01678-w (PMC10923976; doi:10.1007/s10875-024-01678-w)
Supplement: Supplementary file 1 — (DOCX 86.5 KB) [file 10875_2024_1678_MOESM1_ESM.docx]

**Supplemental Information**

**Table S1.** Known causes of thymic stromal defects with a non-exhaustive list of their most common co-morbidities reported to date (+).

| Condition | Genetic defect | Inheritance | Thymic aplasia/ hypoplasia | Congenital heart disease | Hypoparathyroidism | Palatal abnormalities | Facial dysmorphisms | Genitourinary abnormalities | Hearing impairment | Ear abnormalities | Coloboma | Choanal atresia | Nail dystrophy  and alopenia | Skeletal abnormalities | References |
| --- | --- | --- | --- | --- | --- | --- | --- | --- | --- | --- | --- | --- | --- | --- | --- |
| 22q11.2DS | Del 22q11.2 | Most *de novo,*  some AD | + | + | + | + | + | + | + | + |  |  |  | + | [19–22] |
| CHARGE syndrome | *CHD7^+/-^* | Most *de novo,* some AD | + | + | + | + | + | + | + | + | + | + |  | + | [23–28] |
| TBX1 deficiency | *TBX1^+/-^* | AD | + | + | + | + | + |  | + |  |  |  |  |  | [29–34] |
| TBX2 deficiency | *TBX2^+/-^* | AD | + | + | + | + | + |  |  |  |  |  |  | + | [35] |
| Partial monosomy 10p | Del 10p13-14 | Most *de novo* | + | + | + | + | + | + | + |  |  |  |  |  | [36–38] |
| 2p11.2  microdeletion | Del 2p11.2 | Most AD, some *de novo* | + | + |  | + |  |  | + | + |  |  |  | + | [39] |
| FOXI3 haplo-insufficiency | *FOXI3^+/-^* | AD | + |  |  |  |  |  | + | + |  |  |  |  | [49,50] |
| Nude SCID | *FOXN1*^-/-^ | AR | + |  |  |  |  |  |  |  |  |  | + |  | [40–42] |
| FOXN1 haplo-insufficiency | *FOXN1*^+/-^ | AD | + |  |  |  |  |  |  |  |  |  | + |  | [42–44] |
| OFCS2 | *PAX1^-/-^* | AR | + | + | + |  | + |  | + | + |  |  |  | + | [45–48] |
| Maternal diabetes | NA | NA | + | + | + | + | + | + | + | + |  |  |  | + | [18,51,52] |

22q11.2DS, 22q11.2 deletion syndrome; AD, autosomal dominant; AR, autosomal recessive; Del, deletion; FOX, Forkhead Box; NA, not applicable; OFCS2, otofaciocervical syndrome type 2; PAX, Paired Box; SCID, severe combined immunodeficiency; TBX, T-Box Transcription Factor.

**Table S2.** Questions provided as guidance both in email communication and group sessions

| Q1 | *If you think back to when you were first told your child had a suspected immunodeficiency, do you remember how this was explained to you?* |
| --- | --- |
| Q2 | *How quickly were treatment options mentioned, i.e. bone marrow transplantation or thymus transplantation?* |
| Q3 | *How easy was it for you to understand what congenital athymia is, once it was mentioned?* |
| Q4 | *What information sources do you think could have improved your experience at the time of diagnosis?* |
| Q5 | *Do you think NBS positively contributed to an early diagnosis?* |

**Table 3S.** Comprehensive list of parental quotes organised by major reoccurring themes and subthemes

| Theme: Initial newborn screening (NBS) results | |
| --- | --- |
| Benefits | “NBS was very important, not so much in order to have a clear diagnosis very fast, but to take the necessary measures, like isolation”  “If (our child) hadn’t had that NBS, we probably would not be here now”  “I wouldn’t have taken our kids out of care, they would have been in day care and all over (them), I would have been still trying to breastfeed and would have given him CMV by now, (they) would have been a lot sicker”  “We were so lucky to be in that study and we are so grateful”  “(our child) is still infection-free and I think it wouldn’t be possible if we were not isolated”  “That was key”  “Absolutely, we were lucky, not all communities have it”  “Newborn screening has led to early diagnosis and treatment and has protected us during the Corona period”  “We were at the hospital and nurses and doctors and us parents were able to protect our son sooner, plan the treatments sooner and we were able to get a transplant at 3 months age – which was super! A lifesaving test really ☺” |
| Explanations | “The clinicians (neonatologist) said this is maybe because she is premature, it’s probably nothing”  “The doctor (immunologist) didn’t really go into much detail about anything, he was kind of like, everything probably will be ok, it’s probably just an error”  “We have to check if it’s right or not…”  “I was told he has flagged on the NBS for SCID, this is a severe immunodeficiency and you may know it as bubble boy disease” “You know the Bubble child” “For us, it was important to focus on one problem at a time ... They (immunologist) made a roadmap for us”  “After we were first told about the SCID we got little to none information how the future would be like, would our son die, how soon, is it treatable. We were confused and had to Google for information.” |
| Parental understanding | “They (neonatologist) told me like I should know what T cells are … I had no idea”  “I asked what are you saying? I don’t know what you are saying to me? But they (neonatologist) didn’t give me more information and we googled ‘what does it mean when a child doesn’t have lymphocytes’”  “After the phone call, I typed into Google immune problems in newborn screening, and it came up with SCID, so I was reading about it and you know the first thing that you read is about bone marrow transplantation, and really I didn’t know what bone marrow transplantation was, but when you hear the word transplantation, you’re like oh my god and then it says, babies can’t survive”  “At the time we were totally overwhelmed with everything”  “It made no sense”  “I wish they (neonatologist) could have prepared us a little bit more for what it means when these results are not fully ok” |
| Theme: Differential Diagnosis | |
| Severe combined immunodeficiency (SCID) diagnosis | “By the time they said the results were not good, they (neonatologist) were talking about SCID mostly”  “I think I remember him (immunologist) talking about it (SCID) briefly, but it was all kind of gobbledegook, like I had no idea what had caused it”  “They (neonatologist) came in with a piece of paper printed out and it just had SCID information, that was it”  “The first time we heard about the immunodeficiency was after the newborn screening and our doctor (neonatologist) told us our child had a SCID-level immunodeficiency”  “They (immunologist) mentioned both possibilities, but they said that probably the SCID thing is more likely than the other diagnosis”  “It was made clear at this point that further tests were necessary in order to try to narrow down the diagnosis”  “At that point thymus was never on the cards” |
| Bone marrow transplantation | “We were also talking about bone marrow transplantation and everything … we can (tissue type) test everyone in the family (to find a donor)"  “We were told (our child) will need a bone marrow transplantation, we all were immediately sent for tissue typing”)  “After 12 days, we were told that (our baby) would need chemotherapy and stem cell therapy” |
| Congenital athymia diagnosis and/or thymus transplantation | “On the day of the genetic diagnosis two doctors (neonatologist) came in, and were like OK, your child has DiGeorge Syndrome and there is a therapy for it … You have to go to London for a thymus transplant … We were actually totally surprised as nobody really had talked about it before”  “Thymus came weeks after”  “After 9 weeks, a thymus transplant in London was mentioned as an alternative to chemo and stem cell therapy for us”  “Later after the (heart) surgery, as there was no thymus found, we were told that thymus transplant was the best option for us, but we also got our baby’s siblings and us (tissue type) tested for a possibility of a bone marrow transplant”  “What about a thymus? And they (immunologist) were like no, it won’t be a thymus (transplant), he will definitely need a bone marrow transplant, and we were like why’s that? We’ve done a FISH assay and it hasn’t come back DiGeorge. I was very much at that stage, in the very early stages, I’m going to listen to the doctors… you tell us what to do, we trust you” |
| Transparency with information provided | “For me, it was still SCID and the doctors are probably already thinking athymia but they didn’t tell us … We were really surprised as no one really talked about it … That was not the diagnosis we were prepared for”  “Congenital athymia would have come weeks after that (NBS result), it may have been available sooner, but it certainly wasn’t available for us as a family at that point in time, I would say that that was kept behind closed doors with Immunology while they (neonatologist) were looking at solutions”  “maybe it would have been better to kinda have been open about it, to say, … it also might be this … It would have been good to have been a little more prepared”  “I would have loved being told up front, no he doesn’t have SCID, he has congenital athymia, and this is the difference. Because I spent weeks, I wasted weeks learning about SCID, and how am I going to get this kid a bone marrow transplant when none of us match him, and I spent weeks thinking about it, to all of a sudden, no hang on wait, this has totally changed, and we need to now look in this direction because this is our only true option”  “I wish they (neonatologist) were a bit more forward with, hey look, we don’t have that much knowledge on this, and we know there’s knowledge out there, maybe they didn’t know if there was knowledge out there, maybe they didn’t know themselves what (immunologist) would have to share with us … you don’t know what they held onto and what they previously knew before disclosing it to you”  “I didn’t realise that genetics hadn’t been done properly” |
| Quality of information delivered | “It was well explained by the doctor in charge (immunologist), she took the necessary time to get us through the explanations”  “For us, it was important to focus on one problem at a time ... They (immunologist) made a roadmap for us”  “I think he (immunologist) was trying to explain it but it wasn’t like I understood, I didn’t understand anything, it was all like big scientific words. I’m not very good at taking information in, I have to have someone really sit down and explain things to me”  “At the time we were totally overwhelmed with everything”  “they (immunologist) did a great job, they explained us everything they could in a very transparent way, when they didn’t know the reason, or they still don’t why, and were honest enough to tell us, even if we are not 100%”  “At the time I remember (our child) being so fragile … I just remember it unravelling … because we had other diagnoses before … It’s so hard to know how you would like the news delivered to you, when you don’t want it in the first place, but on the flip side once you’ve been there and you’ve done that, and you look back and you think oh yeah, it was a really hard time, what would I have liked put in my hands then and there … Information, just the crucial stuff … This is going to sound so basic, I got handed an A4 piece of paper about SCID, I would have loved having the same about congenital athymia”  “… and I wish when we got told, right, OK he might have this thymus problem, here is something, like a leaflet, something from another parent’s perspective”  “We had a meeting with an immunologist after a few weeks. First, we had to go through our (child’s) heart surgery to plan more. The outcome of the heart surgery would affect which treatment or none the doctors recommended” |
| Theme: Diagnostic Pathway in genetically undefined T lymphocytopaenia | |
| Team collaboration | “They worked together well”  “They always kept us updated”  “We understand what this investigation is”. |
| Access to diagnostics | “I think if I could find this information (diagnostic testing), it should have been considered more and it should have been discussed as a possibility with us, instead of a straight no… I guess it comes down to enough scientific backing” |
| Challenges | “There were months of uncertainty … Doctors (immunologists) were uncertain … So, without a gene, what are our choices … will (our child) reach (their) first birthday?”  “We were waiting for ten weeks for information, and it’s a really long time and you sit there in the hospital, and you don’t know anything and think, what are they waiting for? … We felt forgotten, lost in Googleland” |
| Benefits | “I know (my child) doesn’t have SCID, but (my child) got this thymus problem … It was investigated further and then they (Thymus Transplant team) found out (my child) had a genetic problem, but then they also found out that I have it too, and it’s like I have been fighting my whole life for answers” |
| Theme: Availability of information on athymia and thymus transplantation | |
| Internet searches | “We informed ourselves via Google”  “I typed into Google …  “It was a piece of paper explaining really briefly what thymus transplantation was, but that was about it. Everything else we had to do our own learning around, we had to rely on Google and that, which was so unreliable’”  “Information about SCID and bone marrow transplantation is easy to understand …, but the thymus transplant information, about this, it is not easy to find on the internet, Google”  “We felt forgotten, lost in Googleland”  “We were confused and had to Google for information” |
| Difference between experienced and naïve clinicians to congenital athymia | “They (immunologist) did a great job, they explained us everything they could in a very transparent way, when they didn’t know the reason, or they still don’t why, they were honest enough to tell us …”  “We talk with (our immunologist) and he knows about what to do”  “The moment we received the diagnosis I would say we had thousands of questions, but the doctors (neonatologist) couldn’t answer them”  “It was like, almost don’t ask too many questions, because we (neonatology) need this meeting to happen (with Immunology doctor) where answers will be more free flowing and less of, let’s go back and forth, back and forth all the time”  “(child) was there first SCID kid in 12 years ... they (primary immunology service) did not see a lot of SCID kids”  “After meeting the immunologist we got a little hopeful and understood what that really meant” |
| Information following Immunologist or GOSH contact | “They (neonatologist) also offered to go to (other hospital) to see (the immunology specialist) who was our contact person, which we did after a few days”  “They (Thymus Transplantation team) kind of explained everything a lot better really, they knew exactly what they were talking about, I felt a lot better after I spoke to them”  “After meeting the immunologist, we got a little hopeful and understood what that really meant”  “Once we got to London, they (Thymus Transplantation team) answered all our questions” |
| Theme: Access to support networks | |
| Families | “We felt so alone, so unique, so different from all the other parents I knew … it was great… I remember writing with her as I was staying in (hospital) and (her child) had the transplant a year before us, so she knew a lot and it was great having a person speaking the language I speak”  “We do speak a lot, and I am glad that I have got her … I am lucky to have that, and I know a lot of other people probably don’t have that”  “That would have been a good thing if we had known that there might be other parents who already went through the process, because that’s a different perspective than the perspective of the medical teams”  “I don’t know why I had reservations in the beginning. I think I didn’t know what to ask, and I think maybe that’s because I didn’t get given much information about anything, that I was like ah, I’m just going to have to suck it up and do it, and now I think back and why did I do that, I should have contacted someone and asked questions”  “… and I wish when we got told, right, OK he might have this thymus problem, here is something, like a leaflet, something from another parent’s perspective” |
| Social media | “I found a contact on Instagram to (another parent) and she told me about Facebook groups and thymus transplant support group and the SCID Angels for Life group and there is a lot of information that helped us so much”  “Websites on the internet, contact with other parents, more Facebook groups but more official”  “I’m going to email anyone I can”  “it would be more helpful if we had this information earlier (Facebook group)” |
| Role of clinical nurse specialists (CNS) | “I also wrote a lot with (CNS) … at the time we had (our doctor) but we didn’t have this relationship that I felt always comfortable asking thousands of questions every time I had them”  “I was always contacting (our CNS), constantly… she was really, really good”  “they do not have an Immunology nurse, which I think an Immunology nurse runs the show … I genuinely think you do … (CNS) who we have here, is incredible, absolutely amazing and they do all the background work … I don’t think you can do without an immunology clinical care nurse” |
